# Effects of patient-specific treatment planning on eligibility for photodynamic therapy of deep tissue abscess cavities: retrospective Monte Carlo simulation study

**DOI:** 10.1117/1.JBO.27.8.083007

**Published:** 2022-02-11

**Authors:** Zihao Li, Lam Nguyen, David A. Bass, Timothy M. Baran

**Affiliations:** a University of Rochester, The Institute of Optics, Rochester, New York, United States; b University of Rochester, Department of Biomedical Engineering, Rochester, New York, United States; c University of Rochester Medical Center, Department of Imaging Sciences, Rochester, New York, United States

**Keywords:** Monte Carlo simulation, antimicrobial photodynamic therapy, optical properties, treatment planning, abscess, cavity

## Abstract

**Significance:**

Antimicrobial photodynamic therapy (PDT) effectively kills bacterial strains found in deep tissue abscess cavities. PDT response hinges on multiple factors, including light dose, which depends on patient optical properties.

**Aim:**

Computed tomography images for 60 abscess drainage subjects were segmented and used for Monte Carlo (MC) simulation. We evaluated effects of optical properties and abscess morphology on PDT eligibility and generated treatment plans.

**Approach:**

A range of abscess wall absorptions ($\mu_{a,\text{wall}}$) and intra-cavity Intralipid concentrations were simulated. At each combination, the threshold optical power and optimal Intralipid concentration were found for a fluence rate target, with subjects being eligible for PDT if the target was attainable with <2000  mW of source light. Further simulations were performed with absorption within the cavity ($\mu_{a,\text{cavity}}$).

**Results:**

Patient-specific treatment planning substantially increased the number of subjects expected to achieve an efficacious light dose for antimicrobial PDT, especially with Intralipid modification. The threshold optical power and optimal Intralipid concentration increased with increasing $\mu_{a,\text{wall}}$ (p<0.001). PDT eligibility improved with patient-specific treatment planning (p<0.0001). With $\mu_{a,\text{wall}}=0.2\;{\mathrm{cm}}^{-1}$, eligibility increased from 42% to 92%. Increasing $\mu_{a,\text{cavity}}$ reduced PDT eligibility (p<0.0001); modifying the delivered optical power had the greatest impact in this case.

**Conclusions:**

MC-based treatment planning greatly increases eligibility for PDT of abscess cavities.

## 1 Introduction

Abscesses form as a result of the digestion of solid tissue by bacteria, followed by subsequent envelopment of the localized infection by the host immune system. This results in the formation of a collection of purulent fluid, surrounded by a fibrous capsule. Although this prevents immediate spread of the infection, this encapsulation can prevent further immune involvement and lead to unchecked microbial reproduction. For abscesses that form in the abdomen, the rate of mortality can be as high as 100% when left untreated.^1^ This has led to the development of image-guided percutaneous drainage, which involves the placement of a drainage catheter in the abscess and administration of systemic antibiotics.^2^ Although percutaneous drainage is generally safe and efficacious, therapeutic response can vary widely by patient. Particularly for abscesses that are complex or loculated, cure rates can be as low as 30%.^3^ Abscesses involving pancreatic processes are especially challenging and are more likely to require open surgical drainage,^4^ which carries a higher risk of morbidity and mortality. Further, many abscesses contain antibiotic-resistant species.^5^^–^^7^ This is of particular concern as the proportion of antibiotic-resistant strains is increasing with time,^8^ and the World Health Organization has stated that antimicrobial resistance is considered an “increasingly serious threat to global public health.”^9^

For these reasons, alternative treatment strategies for abscesses, particularly for those that may not respond to standard of care, are required. One promising approach is photodynamic therapy (PDT), which utilizes the combination of a photosensitizer, visible light, and molecular oxygen to generate reactive oxygen species.^10^ PDT has been widely applied for the treatment of cancer,^11^ and has been studied extensively for antimicrobial applications.^12^^,^^13^ We have previously shown that PDT with the photosensitizer methylene blue (MB) is effective at killing multiple bacterial species present in abscess cavities.^7^^,^^14^

With the exception of a single case study in sheep,^15^ animal models of PDT in deep-tissue abscesses are sparse. Therefore, we have initiated a phase 1 clinical trial examining the safety and feasibility of MB-PDT at the time of abscess drainage (ClincalTrials.gov Identifier: NCT02240498). In this trial, subjects receive an infusion of 1 mg/ml MB after completion of the standard of care percutaneous drainage. Following a 10 min incubation period, MB is aspirated, and the cavity is flushed with sterile saline. Intralipid (Fresenius Kabi AG, Bad Homburg, Germany) is then instilled at a concentration of 1%, and a sterile optical fiber is advanced into the approximate center of the cavity. Treatment light at 665 nm is delivered by a laser source (ML7710-PDT, Modulight, Inc., Tampere, Finland), with a target fluence rate of $20\;\mathrm{mW}/{\mathrm{cm}}^{2}$ at the point on the abscess wall closest to the fiber tip. The light dose is escalated using a 3+3 design,^16^ with the initial cohort having a 5 min illumination duration and subsequent groups being escalated in steps of five minutes. In a previous Monte Carlo (MC) study, we estimated that ∼40% of abscess patients treated at our institution would be eligible for MB-PDT.^17^ However, this prior MC study assumed that the uniform dose applied in the current phase 1 trial would be prescribed to all potential subjects.

It has been previously reported that the response to PDT is largely dependent on two main factors: absorbed light dose and photosensitizer concentration.^18^ However, many antimicrobial PDT studies have focused on superficial cases^19^^–^^22^ in which the determination of light dose is greatly simplified compared with the case of deep tissue cavities. In the case of hollow cavities, the integrating sphere effect can greatly increase the fluence rate at the wall of the cavity.^23^ Quon et al.^24^ observed this for the case of the oropharynx, in which a five-fold buildup was noted in the fluence rate. This is also apparent in the nasopharynx, for which van Doeveren et al.^25^ demonstrated a significant fluence rate buildup in three-dimensional (3D) printed phantoms. This study also developed simplified models for treatment planning in hollow cavities. However, these simplified models did not incorporate varying optical properties or fully accurate models of light propagation. Lilge et al.^26^ extended this to bladder cancer, for which they demonstrated the importance of irradiance monitoring on successful delivery of a prescribed light dose. Further, these authors developed MC models of the bladder geometry and found that irradiance was highly dependent on bladder shape and volume. This highlights the need for careful treatment planning in cavities, particularly when tissue optical properties are unknown.

MC simulation provides an ideal method for studying the effects of cavity geometry and optical properties on the light dose delivered to the cavity wall. For many decades, MC has been considered the gold standard for determining the propagation of light through turbid media.^27^ Of particular relevance to the current study, MC can accurately model regions with low or no absorption, and its accuracy is not limited to particular source geometries or distances from the source, unlike analytical approximations.^28^ Crucially, simulations can be performed in parallel using graphics processing units (GPU), which brings MC to near real-time performance. The simulation framework described in the present study builds upon the rich tradition of open-source MC code, incorporating elements from the MCML^29^ and CUDAMCML^30^ software packages. MC has been previously used to perform treatment planning for PDT in oncology,^31^^,^^32^ including by our group.^33^ Although we have previously used MC simulation to examine eligibility for MB-PDT of abscesses, this prior study did not include the effects of changing optical properties or patient-specific treatment plans.^17^

Here, we focus on the generation of patient-specific treatment plans for PDT of abscess patients previously treated at our institution. We examine the effects of absorption at the abscess wall, due to both native tissue optical properties and the addition of MB. By modifying the concentration of Intralipid within the cavity and the optical power delivered, we generate patient-specific treatment plans that aim to achieve a target fluence rate at the abscess wall while minimizing the delivered optical power. We hypothesize that this will increase eligibility for MB-PDT compared with our previous study,^17^ while minimizing risk to potential subjects. Further, we investigate the effects of absorption within the cavity, corresponding to leakage of MB into the cavity following aspiration. We hypothesize that increasing absorption in the cavity will increase the optical power required to achieve the fluence rate target.

## 2 Methods

### 2.1 Study Population

Potential subjects were identified by a retrospective search of the picture archiving and communication system (PACS) at the University of Rochester Medical Center over the time period of January 1, 2014, to December 31, 2014. Inclusion criteria were (1) percutaneous abscess drainage performed at our institution, (2) availability of computed tomography (CT) imaging performed no more than one week prior to drainage, and (3) age≥18  years. Exclusion criteria were (1) presence of more than one abscess cavity and (2) abscess diameter>8  cm. Although some abscesses are imaged with ultrasound, CT imaging was required for the image segmentation described below. Exclusion criteria were chosen to match those in our ongoing phase 1 clinical trial (ClinicalTrials.gov Identifier: NCT02240498). This resulted in 358 possible subjects, of which 60 were chosen at random for further analysis. Due to the retrospective nature of the study, informed consent was waived. All activities were approved by the Research Subjects Review Board at the University of Rochester Medical Center.

### 2.2 Imaging and Image Processing

As described in Sec. 2.1, all subjects received CT imaging prior to abscess drainage to verify the presence of the abscess. These images were de-identified in the PACS, downloaded to an encrypted workstation, and anonymized using the DicomAnonymizerTool.^34^ To aid in identification of the abscess volume, the radiologist’s report for each CT stack was also downloaded from the PACS. This report includes the approximate size and location of the abscess and allows for segmentation to be performed by non-clinical personnel. Each CT stack was then manually segmented to identify the abscess volume using Amira (v2020.2, ThermoFisher Scientific, Waltham, Massachusetts, United States). This was done on a slice-by-slice basis for each subject’s images, with the abscess identified as a region of low enhancement surrounded by a highly enhancing rim. The 3D abscess volume was then exported as a binary DICOM stack for incorporation into MC simulations.

### 2.3 Monte Carlo Simulation

MC simulation was used along with the segmented CT images described above to study light delivery to the cavity wall over a range of optical property assumptions. We used a custom voxel-based MC software package, which has been described previously,^35^ that utilizes graphics processing unit acceleration. This software incorporates elements from the open-source MCML^29^ and CUDAMCML^30^ software packages, while allowing for a dynamic environment including patient-specific 3D geometry and locally varying optical properties. All simulations were run on a Quadro RTX6000 GPU (Nvidia Corporation, Santa Clara, California).

The abscess samples described above were divided into three regions: the abscess wall, inside the abscess, and environment outside the abscess. We made assumptions of tissue parameters (i.e. absorption coefficient, scattering coefficient, scattering anisotropy, and refractive index) for each simulation, shown in Table 1. Descriptions of these assumptions are provided in the last paragraph of Sec. 2.4. The illumination source was a physically accurate model of the flat-cleaved optical fiber used clinically, with a core diameter of 400  μm, refractive index (n) of 1.46, and numerical aperture of 0.22, delivering 665-nm treatment light. An optical power of 1 mW was delivered through propagation of 1,000,000 photon packets, with the fiber face placed at the center of mass of the abscess cavity. Resulting fluence rate maps were scaled linearly to simulate varying emitted optical power.

**Table 1:** Assumptions of tissue parameters at wavelength of 665 nm.

	Abscess wall	Inside abscess	Surrounding tissue
Absorption coefficient ($\mu_{a}$) (${\mathrm{cm}}^{-1}$)	0.2 to 10	0 to 0.17	0.2
Scattering coefficient ($\mu_{s}$) (${\mathrm{cm}}^{-1}$)	100	0 to 100	100
Anisotropy factor (g)	0.9	0.7	0.9
Refractive index (n)	1.4	1.33	1.4

### 2.4 Conditions Simulated

We examined two main cases: (1) changing abscess wall absorption and Intralipid concentration within the cavity simultaneously, assuming no absorption within the cavity, and (2) increasing absorption within the cavity, while allowing for variation in abscess wall absorption and Intralipid concentration within the cavity.

Because absorption at the abscess wall for our clinical application is likely largely determined by the MB concentration and scattering within the abscess is controlled by Intralipid concentration, the abscess wall absorption and scattering inside the abscess cavities were studied as independent variables. The simulated abscess wall absorption ($\mu_{a,\text{wall}}$) ranged from 0.2 to $1\;{\mathrm{cm}}^{-1}$ in $0.1\;{\mathrm{cm}}^{-1}$ increments and from 2 to $10\;{\mathrm{cm}}^{-1}$ in $2\;{\mathrm{cm}}^{-1}$ increments. The simulated scattering coefficient within the abscess cavity ($\mu_{s,\text{cavity}}$) ranged from 0 to $0.4\;{\mathrm{cm}}^{-1}$ in $0.4\;{\mathrm{cm}}^{-1}$ increments, 2.2 to $8.8\;{\mathrm{cm}}^{-1}$ in $2.2\;{\mathrm{cm}}^{-1}$ increments, and 11.1 to $100\;{\mathrm{cm}}^{-1}$ in $11.1\;{\mathrm{cm}}^{-1}$ increments. Simulations were run at each combination of $\mu_{a,wall}$ and $\mu_{s,\text{cavity}}$ for each subject, resulting in a total of 225 simulations for each of the 60 subjects.

For the second case, we allowed absorption within the cavity to increase. In previous studies,^17^ we assumed zero absorption within the abscess cavity. However, a small amount of MB could leak into the abscess, causing the absorption coefficient inside the abscess to be slightly higher than zero (0 to 1  μM MB, resulting in $\mu_{a,\text{cavity}}=0$ to $0.17\;{\mathrm{cm}}^{-1}$). To account for this potential issue, simulations with absorption inside the abscess along with absorption at the abscess wall and scattering inside the abscess were also explored. With three varying parameters, we decided to first simulate a range of $\mu_{a,\text{cavity}}$ at the pre-determined threshold optical power and optimal Intralipid concentration for each abscess cavity, as described below. This represents a scenario in which treatment planning was performed without knowledge of absorption within the cavity and quantifies the effects of $\mu_{a,\text{cavity}}$ on these treatment plans. Next, simulations were performed over a limited range of combinations of abscess wall absorption (0.2 to $1\;{\mathrm{cm}}^{-1}$), scattering within the abscess cavity (0 to $8.8\;{\mathrm{cm}}^{-1}$), and absorption inside the abscess (0 to $0.17\;{\mathrm{cm}}^{-1}$), resulting in 315 simulations for each of the 60 subjects. This corresponds to a scenario in which optical properties are known, both at the abscess wall and within the cavity, allowing an appropriate treatment plan to be generated. Unlike the case without absorption inside the abscess, we examined a narrower range of possible optical property combinations to reduce computational time and to study values that are more likely to emerge clinically.

The absorption coefficient was set to $0.2\;{\mathrm{cm}}^{-1}$ outside the abscess cavity to mimic typical soft tissue absorption in the wavelength range examined, similar to values previously reported for tissue in the peritoneal cavity.^36^ Absorption at the abscess wall ($\mu_{a,\text{wall}}$) ranged from 0.2 to $1\;{\mathrm{cm}}^{-1}$ to model the influence of varying native tissue optical properties and MB uptake. Within the abscess, $\mu_{a,\text{cavity}}$ ranged from 0 to $0.17\;{\mathrm{cm}}^{-1}$ to account for possible leakage of MB solution into the abscess cavity, which corresponds to MB concentrations of 0 to 1  μM. The scattering coefficient was fixed at $100\;{\mathrm{cm}}^{-1}$ for the region outside the abscess and at the abscess wall and varied from 0 to $100\;{\mathrm{cm}}^{-1}$ within the cavity ($\mu_{s,\text{cavity}}$) to investigate the effects of Intralipid concentration. The scattering coefficient for the abscess wall and surrounding tissue was chosen to mimic values observed for intraperitoneal tissue.^36^ These scattering coefficients within the cavity represent Intralipid concentrations ranging from 0% to 2.3%. The refraction index (n) was set to 1.4 for the region outside the abscess and at the abscess wall, corresponding to soft tissue, and 1.33 within the abscess cavity as dilute Intralipid is composed largely of water. The anisotropy factor (g) inside the cavity was assigned to be 0.7, based on previous results for Intralipid at 665 nm,^37^ and 0.9 at the abscess wall and in surrounding tissue.

### 2.5 Eligibility Criteria and Treatment Planning

The maximum (out-of-fiber) power for our clinical laser (ML7710, Modulight, Inc., Tampere, Finland) is 2000 mW, which determines the upper limit of the attainable fluence rate in a given abscess. The goal for treatment planning was to achieve a $4\;\mathrm{mW}/{\mathrm{cm}}^{2}$ fluence rate at 95% of the abscess wall, based on our preclinical results.^7^ In addition, to avoid thermal damage caused by high fluence rates, an upper limit of 5% of the abscess wall receiving $\geq 400\;\mathrm{mW}/{\mathrm{cm}}^{2}$ was also set in this study. If these treatment targets were obtainable with less than 2000 mW, we considered this a subject that would have been eligible for MB-PDT. The threshold power is then the minimum laser power required to achieve the treatment goal for a given combination of optical properties. For a given $\mu_{a,\text{wall}}$, the optimal Intralipid concentration was defined as the Intralipid concentration corresponding to the simulation that minimized the threshold power at that value of absorption, without interpolation between simulated Intralipid concentrations. So, for a given subject, the treatment plan consisted of an optimal Intralipid concentration and corresponding threshold optical power. These treatment plans were compared with a uniform dose treatment plan, in which the power is optimized while the Intralipid concentration is fixed at 1%. This uniform dose case is what is currently deployed in our phase 1 clinical trial as we do not yet have access to patient-specific optical properties.

### 2.6 Statistical Analysis

Throughout, results are summarized across simulation conditions by mean ± standard deviation. The Friedman test was used to compare threshold optical power and optimal Intralipid concentration across simulation conditions. Differences in eligibility between the full treatment planning and uniform dose cases were compared using the Wilcoxon signed-rank test as eligibility data are paired between these cases. All statistical analysis was performed in MATLAB (R2019b, The Mathworks, Inc., Natick, Massachusetts, United States).

## 3 Results

### 3.1 Threshold Optical Power Varies with Abscess Wall Absorption and Intralipid Concentration Within the Cavity

**Fig. 1:**
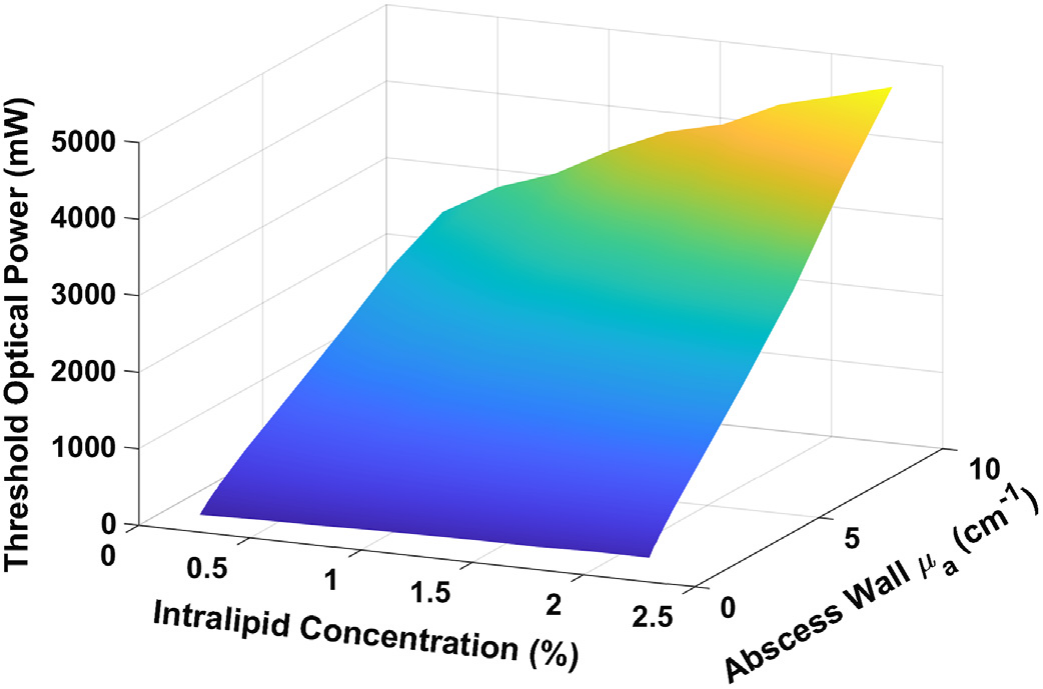
Threshold optical power as a function of abscess wall absorption ($\mu_{a,\text{wall}}$) and Intralipid concentration ($\mu_{s,\text{cavity}}$).

Based on our simulation results, the threshold optical power varied with changes in both abscess wall absorption and intralipid concentration within the cavity. Generally, the threshold optical power increased with both increasing $\mu_{a,\text{wall}}$ and $\mu_{s,\text{cavity}}$. This is shown for a representative pelvic abscess in Fig. 1.

**Fig. 2:**
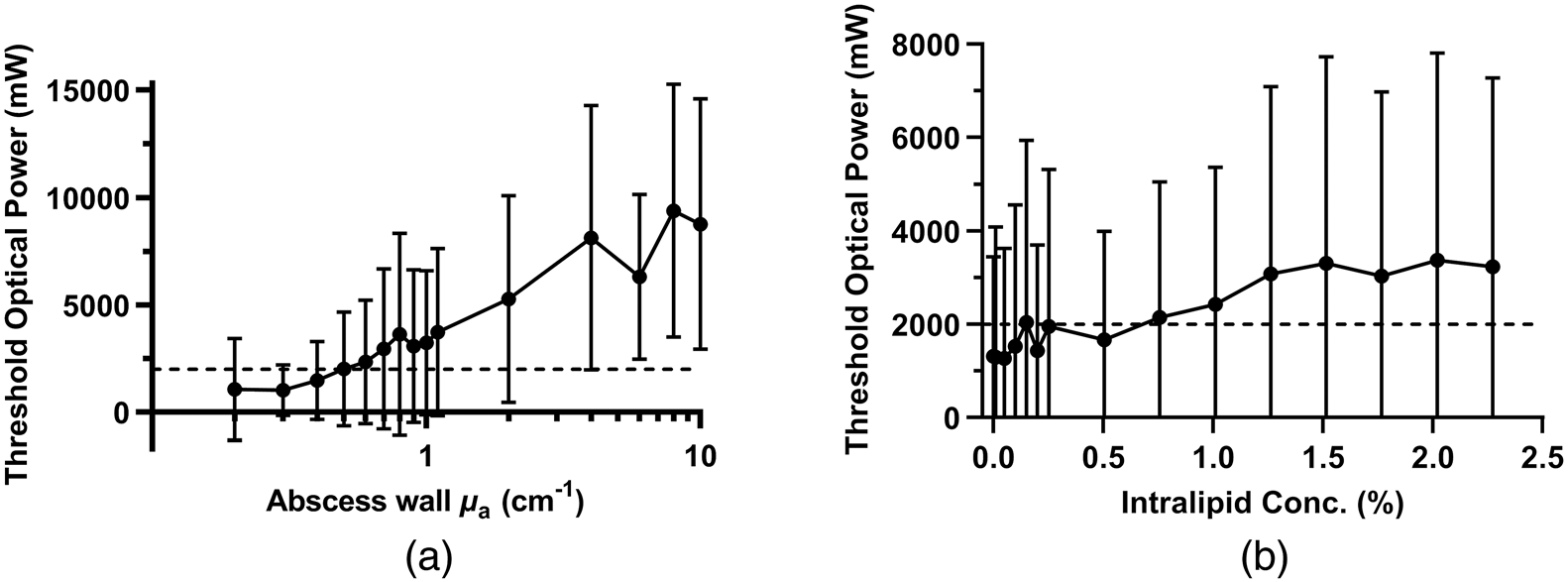
Threshold optical power as a function of (a) abscess wall absorption and (b) Intralipid concentration. The horizontal dashed line indicates the maximum attainable optical power (2000 mW) with our clinical laser. Data points represent mean threshold power across simulations performed for all 60 abscesses, with error bars corresponding to standard deviation.

To quantify these effects, we individually examined the relationships between the threshold optical power and either Intralipid concentration or abscess wall absorption (see Fig. 2). In both cases, the threshold optical power was averaged over all simulations performed at the relevant quantity for all abscesses (e.g., threshold optical power at a given $\mu_{a,\text{wall}}$ was averaged over all $\mu_{s,\text{cavity}}$ values for all 60 abscesses). As shown in Fig. 2(a), the threshold optical power increases with increasing $\mu_{a,\text{wall}}$ across Intralipid concentrations and individual subjects. Applying the Friedman test, this increase was found to be significant (p<0.0001). At higher values of $\mu_{a,\text{wall}}$, many subjects were not eligible for MB-PDT based on the threshold optical power exceeding the 2000 mW limitation established by our clinical laser.

When increasing scattering within the abscess [see Fig. 2(b)], the threshold power shows an increase at high Intralipid concentrations (1% to 2.25%) and a slight increase at low Intralipid concentrations (0% to 1%). Across Intralipid concentrations, this increase was significant (p=0.0005). Due to limitations on maximum clinical laser output (2000 mW), higher Intralipid concentrations (1% to 2.25%) generally lead to ineligibility for MB-PDT.

### 3.2 Optimal Intralipid Concentration is Dependent on Abscess Wall Absorption

**Fig. 3:**
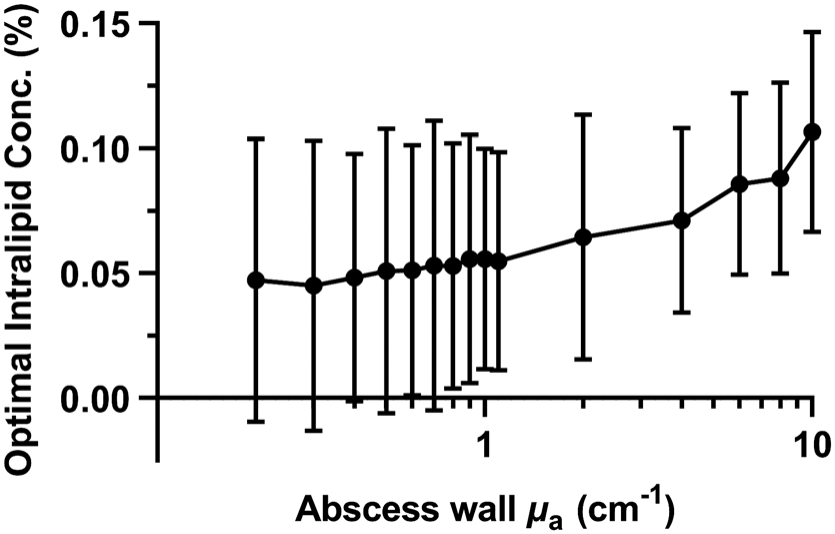
Optimal Intralipid concentration as a function of abscess wall absorption.

The optimal Intralipid concentration is defined as the Intralipid concentration that results in the minimum threshold optical power for a given $\mu_{a,\text{wall}}$. As shown in Fig. 3, we found that this optimal scattering value increased significantly with abscess wall absorption (p<0.0001). For lower values of $\mu_{a,\text{wall}}$ ($<1\;{\mathrm{cm}}^{-1}$), optimal Intralipid concentration did not vary significantly (p=0.74). In this case, the optimal value ranged from 0% to 0.25%. However, at higher abscess wall absorption ($\geq 1\;{\mathrm{cm}}^{-1}$), optimal Intralipid concentration increased substantially (p<0.0001). This highlights the importance of optical property measurements of the abscess wall in individual subjects as the optimal values of the tunable treatment parameters (Intralipid concentration and optical power) are highly dependent on $\mu_{a,\text{wall}}$.

### 3.3 Treatment Planning Greatly Improves Eligibility for Photodynamic Therapy

**Fig. 4:**
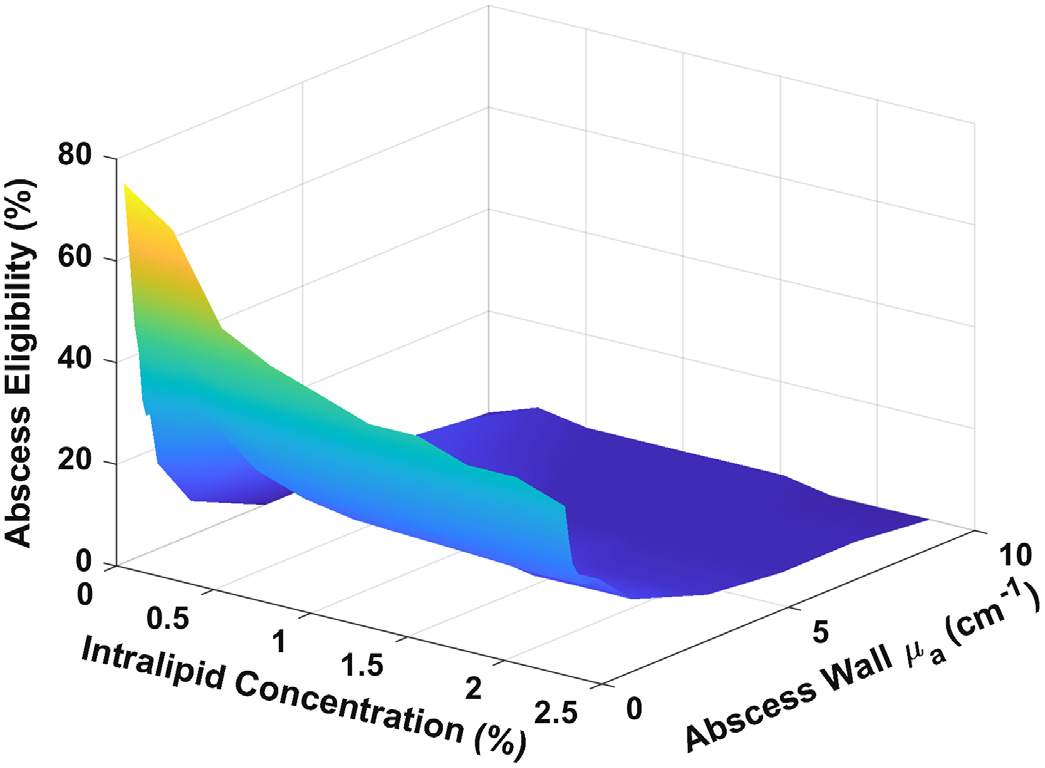
Abscess eligibility for MB-PDT as a function of abscess wall absorption ($\mu_{a,\text{wall}}$) and Intralipid concentration ($\mu_{s,\text{cavity}}$).

We have shown that threshold optical power depends on abscess wall absorption and Intralipid concentration (see Fig. 2), and our definition of abscess eligibility is based on the threshold power, so subject eligibility for MB-PDT here is largely dependent on both abscess wall absorption and Intralipid concentration. As the wall absorption and Intralipid concentration increase, the abscess eligibility generally decreases (see Fig. 4). As mentioned above, eligibility is strongly dependent on $\mu_{a,\text{wall}}$.

**Fig. 5:**
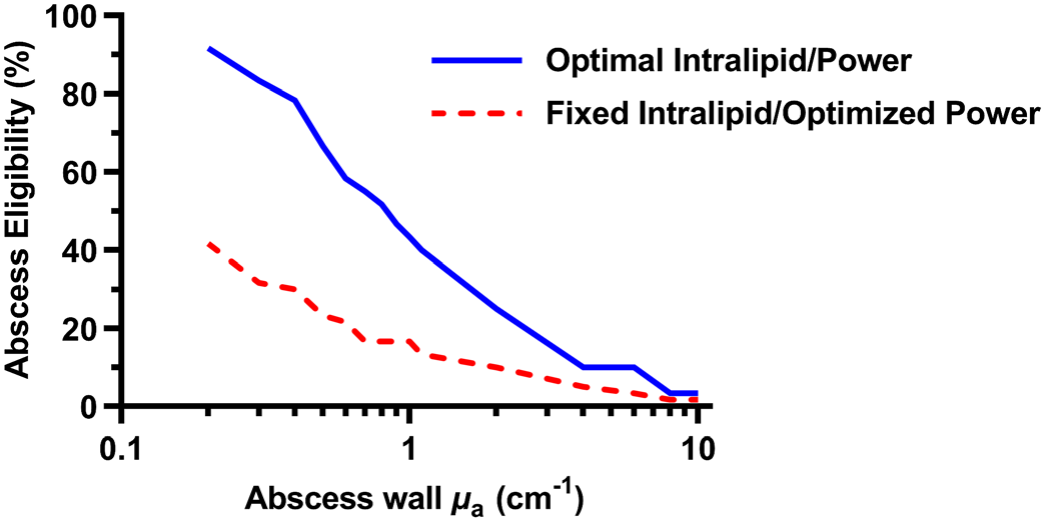
Percentage eligibility for MB-PDT as a function of abscess wall absorption for two treatment methods. Blue is optimized Intralipid concentration and power (patient-specific method). Red is fixed Intralipid concentration with optimized power (uniform dose).

When Intralipid concentration and delivered optical power were optimized simultaneously for each patient, eligibility for MB-PDT increased greatly (Fig. 5). For example, eligibility at $\mu_{a,\text{wall}}=0.2\;{\mathrm{cm}}^{-1}$ increased from 41.7% to 91.7% when patient-specific treatment plans were generated. This increase in eligibility was significant for all values of $\mu_{a,\text{wall}}$ (p<0.0001). Based on these simulation results, we conclude that patient specific treatment planning, with optimization of Intralipid concentration and optical power, could greatly improve eligibility for PDT compared with a uniform dose case in which the Intralipid concentration is fixed. This represents a marked improvement in eligibility compared with the protocol currently employed in our phase 1 clinical trial.

### 3.4 Absorption Within the Cavity Reduces Eligibility and Increases Threshold Optical Power

As described above, leakage of MB into the abscess could result in absorption within the abscess cavity during treatment. If the optical power and Intralipid concentrations were set to those determined for the case of $\mu_{a,\text{cavity}}=0\;{\mathrm{cm}}^{-1}$ when absorption was actually present in the abscess, eligibility was greatly reduced for all cases. Optical pathlengths can be large within the cavity, so any absorption within the cavity greatly reduces the fluence rate at the abscess wall.

**Fig. 6:**
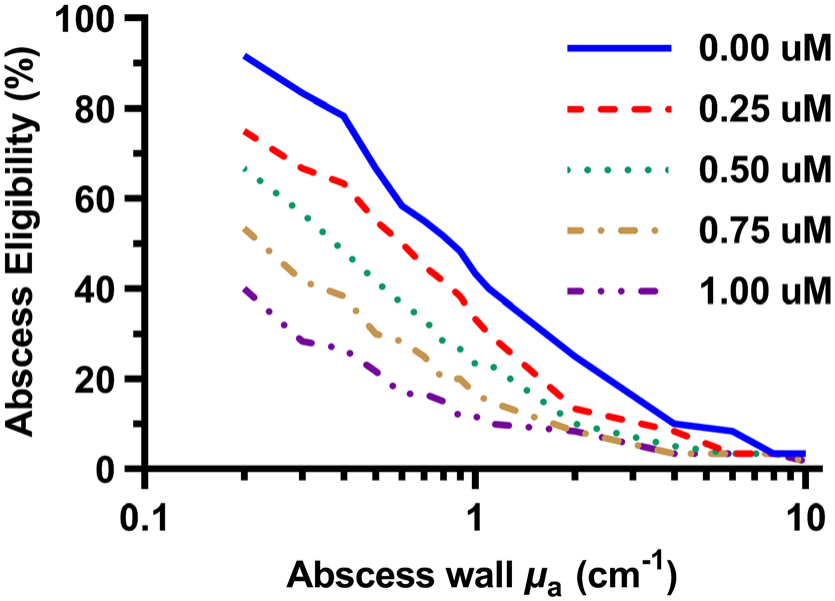
Percentage eligibility for MB-PDT as a function of abscess wall absorption at different levels of absorption inside abscess cavity, corresponding to MB concentrations of 0 to 1  μM, after optimization of delivered optical power.

To overcome this, we performed treatment planning with knowledge of the absorption coefficient inside the cavity. First, we fixed the optimal Intralipid concentration at the value determined for $\mu_{a,\text{cavity}}=0\;{\mathrm{cm}}^{-1}$ and allowed the delivered optical power to vary. Results of this are summarized in Fig. 6. Although there was a significant decrease in eligibility with increasing absorption in the abscess (p<0.0001), these results were significantly better than the case in which optical power and Intralipid concentration were fixed (p<0.0001). We found that threshold optical power increased significantly with increasing absorption inside the abscesses (p<0.001).

**Fig. 7:**
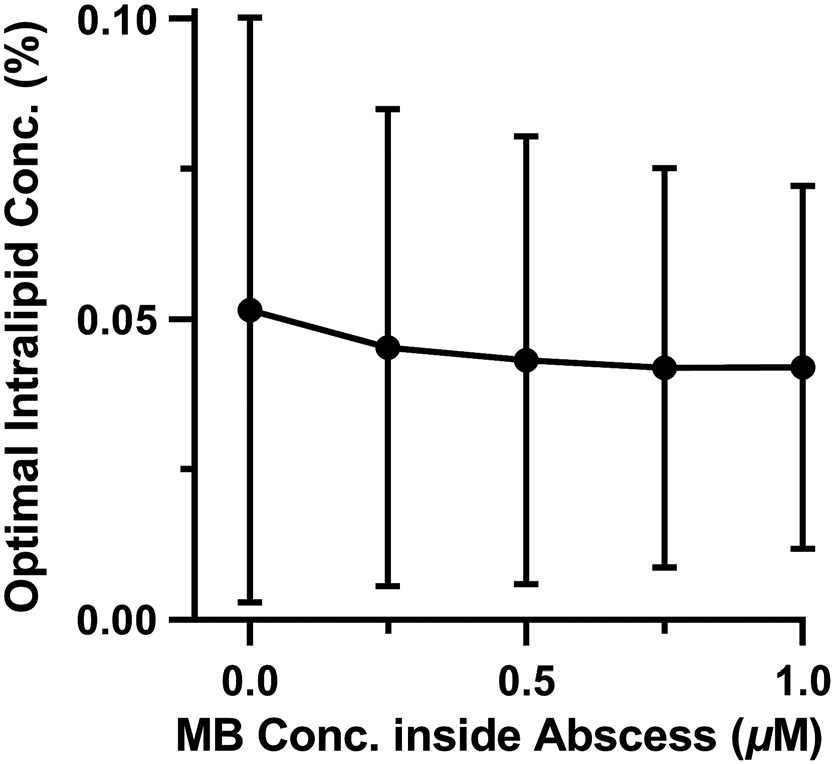
Optimal Intralipid as a function of MB concentration inside the abscess cavity.

Next, Intralipid concentration was also optimized simultaneously with delivered optical power (see Fig. 7). Modification of $\mu_{s,\text{cavity}}$ had a minimal effect on eligibility, particularly for lower values of $\mu_{a,\text{cavity}}$ (p>0.15 in all cases). At the highest value of $\mu_{a,\text{cavity}}$ examined, alteration of Intralipid concentration significantly improved eligibility (p=0.016), though the absolute magnitude of this improvement was small (e.g., 42% versus 40% at $\mu_{a,\text{wall}}=0.2\;{\mathrm{cm}}^{-1}$). Adjustment of the delivered optical power is therefore the main factor driving this recovery of eligibility in the face of increasing $\mu_{a,\text{cavity}}$.

## 4 Discussion

We have demonstrated that eligibility for MB-PDT of abscesses was greatly increased when individual treatment plans were created with knowledge of abscess wall absorption. The power required to achieve a treatment target of $4\;\mathrm{mW}/{\mathrm{cm}}^{2}$ in 95% of the abscess wall was dependent on both absorption at the abscess wall and Intralipid concentration within the cavity, which leads to the determination of an optimal Intralipid concentration minimizing the necessary optical power. This optimal Intralipid concentration increased with increasing abscess wall absorption, particularly for absorption coefficients $>1\;{\mathrm{cm}}^{-1}$. In the case in which absorption was present within the abscess cavity, eligibility for MB-PDT decreased greatly if the optical power and Intralipid concentration were not adjusted. When these factors were optimized with knowledge of absorption inside the cavity, some eligibility was recovered, particularly at lower absorption values.

These results highlight the importance of careful patient-specific treatment planning for PDT. This has been thoroughly investigated for PDT of cancer, for which multiple investigators have reported treatment planning for sites including head and neck,^33^^,^^38^^,^^39^ brain,^40^^–^^42^ and prostate^43^^–^^46^ tumors. Largely in the context of interstitial applications, these previous treatment plans focused on determination of the optimal type and number of source optical fibers, as well as the placement and optical power delivered by these fibers. However, antimicrobial PDT has not seen the same level of investigation in treatment plan development. For superficial applications, this may not be necessary as the treatment field can be directly visualized and the surrounding anatomy does not present a high risk for overtreatment. PDT of deep tissue abscesses, though, is more akin to interstitial oncology applications as the fluence rate cannot be easily measured directly, morphology can vary greatly between patients, and potential damage to surrounding organs is of higher importance. Whereas direct intratumor illumination can require multiple fiber placements to cover the tumor volume (for example, see Altschuler et al.^46^), here we are limited to a single fiber insertion, as dictated by the placement of the standard of care drainage catheter. We, therefore, focus on optimization of the Intralipid concentration within the cavity, as well as the optical power delivered by the source fiber. Although multiple fiber placements may be investigated in the future, the risk of microbial spread due to multiple punctures of the abscess has not yet been established.

Dosimetry and treatment planning for PDT of hollow spaces have been considered by a number of investigators. Perhaps the most prominent have been for PDT of the oropharynx and nasopharynx.^47^^–^^50^ Many of these studies were reviewed by van Doeveren et al.,^25^ with a common feature being the fluence rate buildup that occurs due to the integrating sphere effect, as well as the importance of patient-specific measurements. Researchers at the University of Pennsylvania focused on PDT of mesothelioma within the pleural cavity.^51^ These investigators came to similar conclusions as those above, in that fluence rates are higher at the pleural wall due to the integrating sphere effect and results are highly patient-specific.^52^ One key difference was the necessity for infusion of a scattering solution into the light source and pleural cavity.^53^ Due to the large surface area involved and the non-uniform shape of the tumor bed, this scattering is required to ensure that sufficient light dose is delivered to the entire target region, which also motivates the incorporation of a comprehensive dosimetry system.^53^^,^^54^

**Fig. 8:**
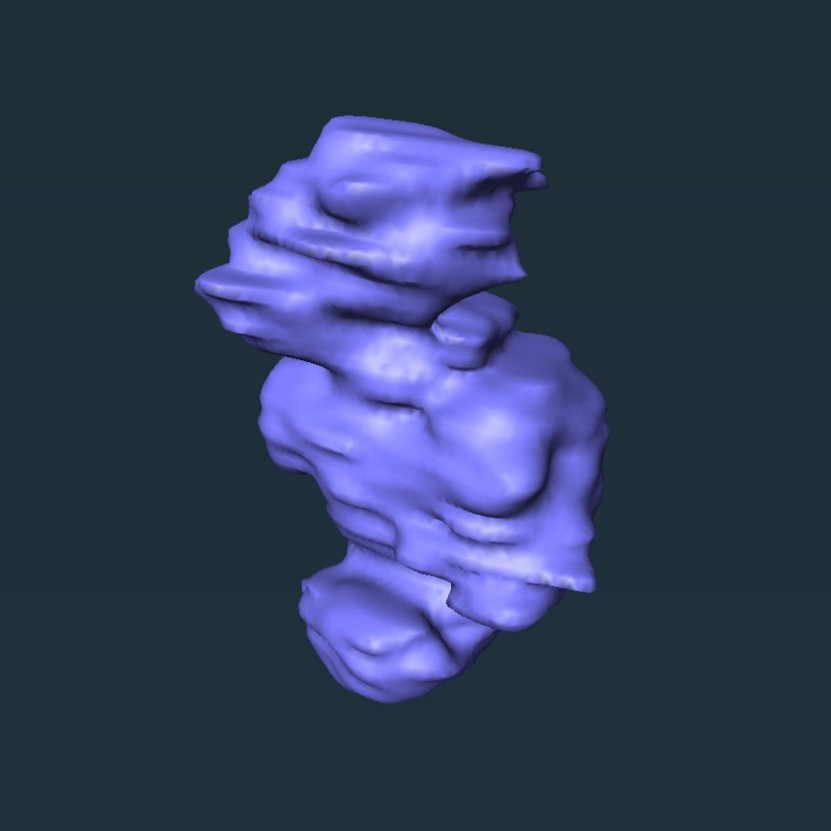
Surface rendering of pelvic abscess in which subject was ineligible for MB-PDT with 0% Intralipid concentration but was eligible at 0.5% Intralipid, assuming $\mu_{a,\text{wall}}=1\;{\mathrm{cm}}^{-1}$.

Lilge et al.^26^ examined the effects of optical properties at the wall, bladder shape, and scattering within the bladder in the context of a phase 1 trial for PDT treatment of bladder cancer. This study demonstrated that the major factors influencing dose were bladder shape and volume, with optical properties at the wall having only a minor effect. Additionally, the authors argue that scattering within the bladder should be heavily reduced or eliminated entirely. On the other hand, we demonstrate here that absorption at the abscess wall has a large effect on the desired optical power and eligibility for MB-PDT (see Figs. 2 and 4). Further, the optimal Intralipid concentration was found to increase with increasing absorption at the abscess wall (see Fig. 3), and was non-zero in many cases even at lower abscess wall absorption. There are a number of possible explanations for this apparent discrepancy between the present study and Lilge et al.^26^ First, abscess shape can be highly irregular as compared with the bladder. The bladder has a defined anatomical position, with modest shape changes caused by factors such as urine flow, tumor growth, or inflammation of surrounding organs.^55^^,^^56^ Abscesses, on the other hand, can develop in arbitrary locations within the abdomen, including formation around the bowel loops or along the abdominal wall.^57^ This can lead to highly non-ellipsoidal shapes, as shown in Fig. 8.

Particularly for these irregular abscesses, increased scattering within the cavity may be required to overcome strong shape effects. For example, for the abscess shown in Fig. 8, this subject would not have been eligible for MB-PDT at $\mu_{a,\text{wall}}=1\;{\mathrm{cm}}^{-1}$ with 0% Intralipid but would have been eligible at 0.5% Intralipid. Second, Lilge et al. examined a relatively smaller range of optical properties than those studied here. This range was appropriate for their application, in which absorption is largely dependent on endogenous chromophores in the bladder wall. In our case, we employ MB as a photosensitizer, which has a high extinction coefficient, even at the diluted concentrations used clinically. Because the amount of MB that is retained by bacteria present at the abscess wall is currently unknown, we cannot assume that absorption at the abscess wall will vary over a small range. Therefore, we examined a large range of potential $\mu_{a}$ values at the abscess wall, corresponding to MB concentrations as high as ∼60  μM. Increases in optimal Intralipid concentration are most apparent at these high $\mu_{a}$ values, as shown in Fig. 3.

The dependence of optimal Intralipid concentration and laser power on absorption at the abscess wall motivates the measurement of abscess wall optical properties in human subjects. Toward this end, we have built and validated an optical spectroscopy system for this purpose. Pre-clinical validation has shown good recovery of both absorption and scattering from tissue simulating phantoms, including cases in which multiple absorbers were present.^58^ This system is similar to other spatially-resolved diffuse reflectance systems that have been employed in the context of treatment planning for PDT and photothermal therapy.^43^^,^^59^^,^^60^ However, our design allows for minimally invasive measurement through the standard of care drainage catheter and does not require assumption of absorber spectral shape. Following full approval, these spectroscopy measurements will be incorporated into the phase 1 clinical trial described above. This will allow for generation of truly patient-specific treatment plans, which we have shown here to greatly expand the number of subjects that we would expect to benefit from MB-PDT.

A strong dependence between absorption within the abscess cavity and eligibility for MB-PDT was also demonstrated here. These results motivate potential collection of the Intralipid solution aspirated from the abscess following clinical MB-PDT. This would allow for quantification of the absorption present within the cavity at the time of therapeutic illumination and could impact the optical power and Intralipid concentration chosen for future treatments. Although our clinical results to date demonstrate excellent recovery of the MB instilled into the cavity (median 98% recovery, 90% to 100% range), small amounts of MB present in the interior could have large effects on the desired treatment plan.

We acknowledge a number of limitations in the study described here. First, uniform optical properties were assumed across the abscess wall. As described above, an optical spectroscopy system is currently being developed to allow for measurement of human abscess wall optical properties. Data on heterogeneity of these optical properties will be incorporated into future work as the simulation framework utilized can accommodate local variations in optical properties. Related to this, uniform MB uptake was assumed, so eligibility was based only on achieving the desired fluence rate in 95% of the abscess wall. Other investigators have proposed that combined drug-light product or reacted singlet oxygen may be better predictors of PDT outcome.^61^^–^^63^ As data are collected on MB uptake heterogeneity, this information will be included in future work to determine whether drug-light is more strongly associated with outcome than the fluence rate alone. Additionally, results presented here are for a subset of abscess patients treated at our institution. Although we have increased the sample size by 50% compared with our previous work,^17^ the conclusions drawn could still be vulnerable to selection bias. Finally, the fluence rate threshold chosen is based on pre-clinical results.^7^ Ongoing studies may establish alternative thresholds, which would affect the exact eligibility proportions reported here. However, conclusions could be readily adjusted using any threshold fluence rates determined.

## Code, Data, and Materials Availability

The data and code underlying the results outlined here are not currently publicly available but may be obtained from the authors upon reasonable request, with the exception of clinical imaging data.
